# Differential Protein Expression Analysis of Two Sugarcane Varieties in Response to Diazotrophic Plant Growth-Promoting Endophyte *Enterobacter roggenkampii* ED5

**DOI:** 10.3389/fpls.2021.727741

**Published:** 2021-11-23

**Authors:** Dao-Jun Guo, Dong-Ping Li, Rajesh Kumar Singh, Pratiksha Singh, Anjney Sharma, Krishan K. Verma, Ying Qin, Qaisar Khan, Zhen Lu, Mukesh K. Malviya, Xiu-Peng Song, Yong-Xiu Xing, Yang-Rui Li

**Affiliations:** ^1^College of Agriculture, Guangxi University, Nanning, China; ^2^Key Laboratory of Sugarcane Biotechnology and Genetic Improvement, Ministry of Agriculture and Rural Affairs, Sugarcane Research Center, Chinese Academy of Agricultural Sciences, Guangxi Key Laboratory of Sugarcane Genetic Improvement, Sugarcane Research Institute, Guangxi Academy of Agricultural Sciences, Nanning, China; ^3^Guangxi Key Laboratory of Crop Genetic Improvement and Biotechnology, Nanning, China; ^4^Microbiology Institute, Guangxi Academy of Agricultural Sciences, Nanning, China

**Keywords:** *Enterobacter roggenkampii* ED5, endophyte, N-fixation, PGP, proteome, sugarcane, TMT

## Abstract

Plant endophytic bacteria have many vital roles in plant growth promotion (PGP), such as nitrogen (N) fixation and resistance to biotic and abiotic stresses. In this study, the seedlings of sugarcane varieties B8 (requires a low concentration of nitrogen for growth) and GT11 (requires a high concentration of nitrogen for growth) were inoculated with endophytic diazotroph *Enterobacter roggenkampii* ED5, which exhibits multiple PGP traits, isolated from sugarcane roots. The results showed that the inoculation with *E. roggenkampii* ED5 promoted the growth of plant significantly in both sugarcane varieties. ^15^N detection at 60 days post-inoculation proved that the inoculation with strain ED5 increased the total nitrogen concentration in the leaf and root than control in both sugarcane varieties, which was higher in B8. Biochemical parameters and phytohormones in leaf were analyzed at 30 and 60 days after the inoculation. The results showed that the inoculation with *E. roggenkampii* ED5 improved the activities of superoxide dismutase (SOD), catalase (CAT), NADH-glutamate dehydrogenase (NADH-GDH), glutamine synthetase (GS), and endo-β-1,4-glucanase, and the contents of proline and indole acetic acid (IAA) in leaf, and it was generally more significant in B8 than in GT11. Tandem Mass Tags (TMT) labeling and liquid chromatography-tandem mass spectrometry (LC-MS/MS) were used to perform comparative proteomic analysis in the sugarcane leaves at 30 days after inoculation with strain ED5. A total of 27,508 proteins were detected, and 378 differentially expressed proteins (DEPs) were found in the treated sugarcane variety B8 (BE) as compared to control (BC), of which 244 were upregulated and 134 were downregulated. In contrast, a total of 177 DEPs were identified in the treated sugarcane variety GT11 (GE) as compared to control (GC), of which 103 were upregulated and 74 were downregulated. The DEPs were associated with nitrogen metabolism, photosynthesis, starch, sucrose metabolism, response to oxidative stress, hydrolase activity, oxidative phosphorylation, glutathione metabolism, phenylpropanoid metabolic process, and response to stresses in Gene Ontology (GO) and Kyoto Encyclopedia of Genes and Genomes (KEGG) database. To the best of our knowledge, this is the first proteomic approach to investigate the molecular basis of the interaction between N-fixing endophytic strain *E. roggenkampii* ED5 and sugarcane.

## Introduction

Sugarcane (*Scacharum* spp. interspecific hybrids) is a major sugar and bioenergy crop worldwide (Li and Yang, 2015; Bordonal et al., 2018; Parsaee et al., 2019). The production of sugarcane requires a large practice of fertilizers, especially nitrogen (N). However, excess N fertilizer contributes to the soil, groundwater, and environmental pollution, as well as high cost for crop production (Basanta et al., 2003; Franco et al., 2011, 2015; Boschiero et al., 2020). Plant growth-promoting bacteria (PGPB) are the microorganisms that can colonize in plant tissues or rhizospheric soil and show beneficial effects on plant development, biotic or abiotic stresses, and nutrient assimilation (Esitken et al., 2010; Piromyou et al., 2011; Orozco-Mosqueda et al., 2020). PGPB promotes the growth of plant through various ways, such as the production of siderophore, solubilization of phosphate, secretion of indole acetic acid (IAA), enhancement of nutrient absorption by plants, and improvement of tolerance to phytopathogens (Lugtenberg and Kamilova, 2009). Some PGPB can convert natural molecular nitrogen into ammonia and provide it to the host plants (Reis and Teixeira, 2015; Igiehon and Babalola, 2018). It is effective to use these N-fixing bacteria to improve nitrogen nutrition in plants which is necessary for crop production, especially in sugarcane (Wei et al., 2014; Singh et al., 2017). At present, some reports are available on the isolation of N-fixing microorganisms with PGP traits from rhizospheric soil or tissues of sugarcane, such as *Klebsiella variicola*, *Kosakonia sacchari*, *Streptomyces chartreusis*, *Pseudomonas* spp., *Herbaspirillum seropedicae*, *Stenotrophomonas maltophilia*, *Kosakonia radicincitans*, *Bacillus* spp., *Pantoea dispersa*, and *Enterobacter asburiae* (Chen et al., 2014; Wang Z. et al., 2016; Li et al., 2017; Antunes et al., 2019; Singh et al., 2020, 2021b).

At present, some progress has been made in studies of PGPB-plant interaction mechanisms to enhance disease resistance, tolerance to abiotic stresses, and N-fixation in plants (Fernandez et al., 2012; Imran, 2013; Sivasakthi et al., 2014; Kumari et al., 2019). Regarding the advantage of genomics, transcriptomics, proteomics, metabolomics, and other molecular biological technologies can reveal the complete mechanism of the interactions between microorganisms and plants (Shen et al., 2013; Xie et al., 2015; Mhlongo et al., 2018). The mechanism of interaction between microorganisms and plants is widely investigated by using proteomic approaches. Recently, protein labeling and liquid chromatography-tandem mass spectrometry (LC-MS/MS) have been widely applied (Chinnasamy, 2006; Kwon et al., 2016; Naher et al., 2018; Bai et al., 2019; Alberton et al., 2020), which is highly efficient in identifying and quantifying differentially expressed proteins (DEPs) in different samples to investigate the mechanisms of salt tolerance (Jiang et al., 2017; Pan et al., 2019; Ma et al., 2020), drought resistance (Xie et al., 2016; Wang Y. et al., 2019; Asghari et al., 2020), and disease resistance (Wang J. et al., 2016; Li et al., 2017; Shi et al., 2019; Singh et al., 2021a) in plants. At present, Tandem Mass Tags (TMT) technology is one of the most powerful analytical approaches for the quantitative analysis of differential proteins in a protein profile and to explain the molecular mechanisms of different experimental treatments (Lü et al., 2016; Zhu et al., 2019; Wu et al., 2020). Earlier, this technique was used to investigate the effect of oxidative stress on the heat tolerance of *Pseudomonas fluorescens* (Chen et al., 2019). For different wheat varieties infected with *Fusarium*, a total of 366 DEPs were identified by TMT technology and the enrichment of the DEPs in phenylpropane biosynthesis, secondary metabolite biosynthesis, glutathione metabolism, signal transduction of phytohormones, mitogen-activated protein kinase (MAPK) signal transduction pathway, photosynthesis was observed, revealing the defense mechanism of wheat against *Fusarium* (Qiao et al., 2021).

According to Guo et al. (2020), *Enterobacter roggenkampii* ED5 isolated from the sugarcane roots exhibited high nitrogenase activity with multiple PGP traits, as well as enhanced sugarcane growth under greenhouse conditions. Analysis of the complete genome sequence of this isolate also confirmed the presence of multiple *PGP* genes in its genome, which might be linked to N-fixation and PGP in sugarcane plants (Guo et al., 2020). However, the molecular mechanisms of the interaction between *E. roggenkampii* ED5 and sugarcane are still unclear.

In this study, the technology of TMT labeling and LC-MS/MS was applied to investigate the comparative protein profiles of the two sugarcane varieties (i.e., B8 and GT11) inoculated with *E. roggenkampii* ED5 under greenhouse conditions. This study aimed to reveal (i) the response to oxidative kinase activity, (ii) the level of plant hormones, (iii) the pattern in nitrogen use, and (iv) the key proteins involved in sugarcane growth promotion after inoculation with *E. roggenkampii* ED5, so as to provide a reference for further study of *E. roggenkampii* ED5 and its application in sugarcane production. To the best of our knowledge, this is the first study to explore the molecular mechanisms of the interaction between *E. roggenkampii* and sugarcane using a proteomic approach.

## Materials and Methods

The strain *E. roggenkampii* ED5 was isolated from the sugarcane roots planted at Sugarcane Germplasm Resource Nursery, Guangxi Academy of Agricultural Sciences (GXAAS), Nanning, Guangxi, China. Two sugarcane varieties with different levels of N-fixation, namely, the variety B8 (needs less nitrogen for growth) and the variety GT11 (needs more nitrogen for growth), were used in the experiment. The field soil was sterilized at 121°C, autoclaved for 30 min, and used for pot experiment. The upper diameter of the pots was 20 cm, bottom 16 cm, and a height of 15 cm. The experimental site was located in the greenhouse of Sugarcane Research Institute, GXAAS, Nanning, Guangxi, China. The enzyme analysis kits were obtained from Suzhou Gres Biotech Co., Ltd., China. The ammonium sulfate-^15^N_2_ isotope label chemical was obtained from the Bioisotope Engineering Center, Shanghai, China.

### Bacterial Culture Conditions and Cultivation of Sugarcane Seedlings

The strain *E. roggenkampii* ED5 was cultured in Luria-Bertani (LB) agar medium and incubated at 32°C and 220 rpm in a shaker (48 h). After incubation, the pellet was collected by centrifugation of bacterial culture at 5,000 × *g* for 15 min and the pellet was resuspended in sterile water to obtain the desired bacterial concentration (10^6^ CFU/ml) for the inoculation in sugarcane. Sugarcane bud setts were placed in a tray, covered with sterilized soil, and watered appropriately, and grown seedlings with uniform size (∼15 cm) were used for the experiment.

### Greenhouse Experiment and Sampling

For pot experiment, 60 healthy sugarcane plantlets of each sugarcane variety were divided into two groups (30 plants in each group). The sugarcane seedlings were uprooted from the tray. Before the seedlings were transplanted into pods, their roots were cleaned properly with running tap water and soaked in a culture broth with a concentration of 10^6^ CFU/ml for 1 h. The seedlings root-soaked with sterile water were used as control. The experiment was conducted in a randomized block design (RBD), and each experiment was repeated thrice (*n* = 3). Sugarcane leaf + 1 (top visible dewlap leaf) samples were collected for analysis at 30 and 60 days after the treatment.

### Total Nitrogen and ^15^N Abundance of the Sugarcane Plant

Kjeldahl method of nitrogen determination was used for total nitrogen test (Sáez-Plaza et al., 2013). The ^15^N isotope dilution method has the potential to measure the BNF in both B8 and GT11 sugarcane varieties. This technique involves inoculating with N-fixing ED5 strain to the sugarcane plants. Soil and sand were blended in a 1:3 (w/w) ratio and sterilized twice for 45 min at 121°C. After cooling at ambient temperature, 10 mg ammonium sulfate-^15^N (10.12% atom ^15^N excess) was applied per kilogram of soil and homogenized to ensure consistent dispersion of (^15^NH_4_)_2_SO_4_. The experiment was conducted according to the method followed by Lin et al. (2012), and nitrogen derived from the air (Ndfa) in the leaf and root of sugarcane tissues was calculated as suggested by Urquiaga et al. (1992).

### Analyses of Biochemical Parameters

The quantitative changes in biochemical parameters related to oxidative stress, namely the activities of superoxide dismutase (SOD), catalase (CAT), and peroxidase (POD) and the contents of malondialdehyde (MDA) and proline (Pro); those related to nitrogen metabolisms, such as the activities of NADH-glutamate dehydrogenase (NADH-GDH), glutamine synthetase (GS), nitrate reductase (NR); and those related to biocontrol mechanisms, such as the activities of β-1,3-glucanase and endo-β-1,4-glucanase, were observed at 30 and 60 days after the treatment. The analyses were performed using Biochemical Test Kit (Suzhou Grace Biotechnology Co., Ltd., China) following the instructions of the manufacturer. The results of all the analyzed biochemical parameters were presented as the ratio of values of the inoculated treatment and control.

### Determination of Phytohormones

The phytohormones in the sugarcane leaf samples were detected at 30 and 60 days after inoculation with strain ED5. Phytohormones, such as, indole-3-acetic acid (IAA), abscisic acid (ABA), and gibberellins (GA_3_), were measured by high-performance liquid chromatography (HPLC) (LC-100 PLUS, Shanghai, China). Notably, 0.2 g of sugarcane leaf + 1 samples were grounded with liquid nitrogen and diluted with 1 ml of precooled methanolic acid solution (70–80%) and kept overnight (4°C) for extraction. The extracts were centrifuged at 6,000 × *g* for 10 min (4°C) and extracted again with 0.5 ml of methanol solution (70–80%) for 2 h (4°C). The supernatants were collected after centrifugation, mixed, and evaporated to one-third volume under reduced pressure at 40°C and then an equal volume of petroleum ether was added for layering. The extraction and decolorization process was repeated 2–3 times. Later, triethylamine was added and the pH was adjusted to 8.0, incubated with shaking at 150 rpm for 20 min after the addition of cross-linked polyvinylpyrrolidone (CTFA). The supernatant was adjusted to pH 3.0 with hydrochloric acid after centrifugation and extraction with ethyl acetate and then evaporated to dry under reduced pressure at 40°C. The mobile-phase solution was added and vortexed to dissolve it, filtering with a syringe filter for measurement. The C18 reversed-phase column (150 mm × 4.6 mm, 5 μm) was used for HPLC at 0.8 ml/min flow rate (25°C). The mobile phase was made up of solvent A and solvent B. Solvent A was 100% methanol and solvent B was 0.1% acetic acid aqueous solution (A:B = 55:45). The injection volume was 20 μl, and the UV wavelength was set at 254 nm.

### Protein Extraction and Tandem Mass Tag Labeling

The Tris-balanced phenol extraction method (BPP method) was used to extract protein from the sugarcane leaves on 30-day inoculated strain ED5. Notably, 0.2 g of sugarcane leaf + 1 samples stored at −80°C in a freezer were transferred into a shaking tube and mixed with the appropriate amount of borax/polyvinyl-polypyrrolidone/phenol (BPP) solution and grounded for six times with a grinder (180 s each time). After centrifugation at 12,000 × *g* for 20 min at 4°C, the supernatant was mixed with an equal volume of Tris-saturated phenol, stored at 4°C for 30 min, and then vortexed for 10 min. Again, the supernatant was centrifuged at 12,000 × *g* for 20 min under 4°C, and the phenol phase was collected and added with an equal volume of BPP solution and then vortexed for 10 min at 4°C. Then supernatant was centrifuged for 20 min at 12,000 × *g* under 4°C. The phenol phase was collected and mixed with five volumes of precooled ammonium acetate methanol solution, and protein was precipitated overnight at −20°C. After incubation in a water bath at 25°C, the extracts were centrifuged at 12,000 × *g* for 20 min under 4°C. The supernatant was discarded and 1 ml 90% precooled acetone was added, mixed well, centrifuged, and again the supernatant was discarded. This step was repeated twice. The precipitates were dissolved in 1 ml protein lysate (8 M urea + 1% sodium dodecyl sulfate, a cocktail containing protease inhibitor) for precipitation and sonicated for 2 min on ice. The mixtures were centrifuged at 12,000 × *g* for 20 min under 4°C to obtain the protein supernatant. Notably, 100 μg of the protein extracts were added with appropriate amount of lysate to 1 ml kept for 60 min in 37°C water bath, and finally 1 ml of 10 mM Tris(2-carboxyethyl)phosphine (protein-reducing agent) was added. Then, 1 ml of 40 mM iodoacetamide was added and reacted in dark at room temperature (RT) for 40 min. Later, 1 ml of precooled acetone was added to each tube, precipitated at 4°C for 4 h, and centrifuged at 10,000 × *g* for 20 min. The precipitates were collected and fully dissolved in 1 ml of 100 nM TEAB, and trypsin was added according to the mass ratio of 1:50 (enzyme:protein), hydrolyzed overnight at 37°C, and then stored in a dry place. Notably, 100 μg of the peptide obtained by enzymatic digestion were added with 1 ml TMT reagent (Thermo Fisher Catalog No. 90111) and incubated at RT for 2 h. Again, 1 ml hydroxylamine was added and kept at RT for 15 min. Equal amount of the TMT-labeled product was mixed in a tube and vacuum-concentrated. The detailed information of all proteins obtained from the three biological replicates of sugarcane leaf + 1 samples is presented in Supplementary Tables 1, 2.

### Proteomic Analysis of High-Performance Liquid Chromatography-Electrospray Tandem Mass Spectrometry

The peptide samples were reconstituted with the one-dimensional ultraperformance liquid chromatography loading buffer, and the reverse phase C18 column (ACQUITY UPLC BEH C18 Column 1.7 μm, 2.1 mm × 150 mm, Waters, United States) was used for high pH liquid-phase separation. Phase A (2% acetonitrile) and phase B (80% acetonitrile) were used for liquid chromatography. A total of 20 fractions were collected according to the peak type and time and combined into 10 fractions. After concentration by vacuum centrifugation, they were dissolved in the mass spectrometry loading buffer (2% acetonitrile and 0.1% formic acid) for the two-dimensional analysis, and the second dimension adopts nanoliter volume flow HPLC liquid system. Easy-nLC1200 was used for separation. The peptides were diluted in mass spectrometry loading buffer and separated by chromatography for 120 min after loading with a volume flow rate of 300 μl/min. EASY-nLC liquid phase underwent gradient elution with phase A 2% acetonitrile (ammonia water, pH 10) and phase B 80% acetonitrile (ammonia water, pH 10). The elution gradients were as follows: 0–1 min, 5% solvent B; 1–63 min, 5–23% solvent B; 63–77 min, 23–29% solvent B; 77–86 min, 29–38% solvent B; 86–88 min, 38–48% solvent B; 88–89 min, 48–100% solvent B; 89–95 min, 100% solvent B; and 95–96 min, 100–0% solvent B.

### Bioinformatics Analysis

Proteome Discoverer, TM Software version 2.1 was used to analyze the original files from the mass spectrometer. The significance *p*-value of the difference between samples was calculated using the *t*-test function in the R language. The screening criterion for significantly DEPs was *p* < 0.05, and the differences > 0.83 and > 1.2 between the two samples indicated downregulated and upregulated proteins, respectively. Molecular functions of the identified DEPs were annotated by Cluster of Orthologous Groups of Proteins (COG), Pfam, Gene Ontology (GO), Kyoto Encyclopedia of Genes and Genomes (KEGG), and Non-Redundant Protein Sequence (NR) databases. The software BLAST2GO version 2.5.0 was used for the enrichment of GO function and KOBAS version 2.1.1 was used for the enrichment of KEGG GO function.

### Data Analysis

Plant biochemical data were analyzed using Excel 2010, and SPSS version 20.0 software was used to perform the analysis of variance (ANOVA). The difference in significance at *p* ≤ 0.05 was used to assess the comparison between the means. The statistical analysis for DEPs was carried out using Majorbio I-Sanger Cloud Platform, a free online platform^1^.

## Results

### Plant Oxidative Stress, Biocontrol, and Nitrogen Metabolism-Related Biochemical Parameters

**FIGURE 1:**
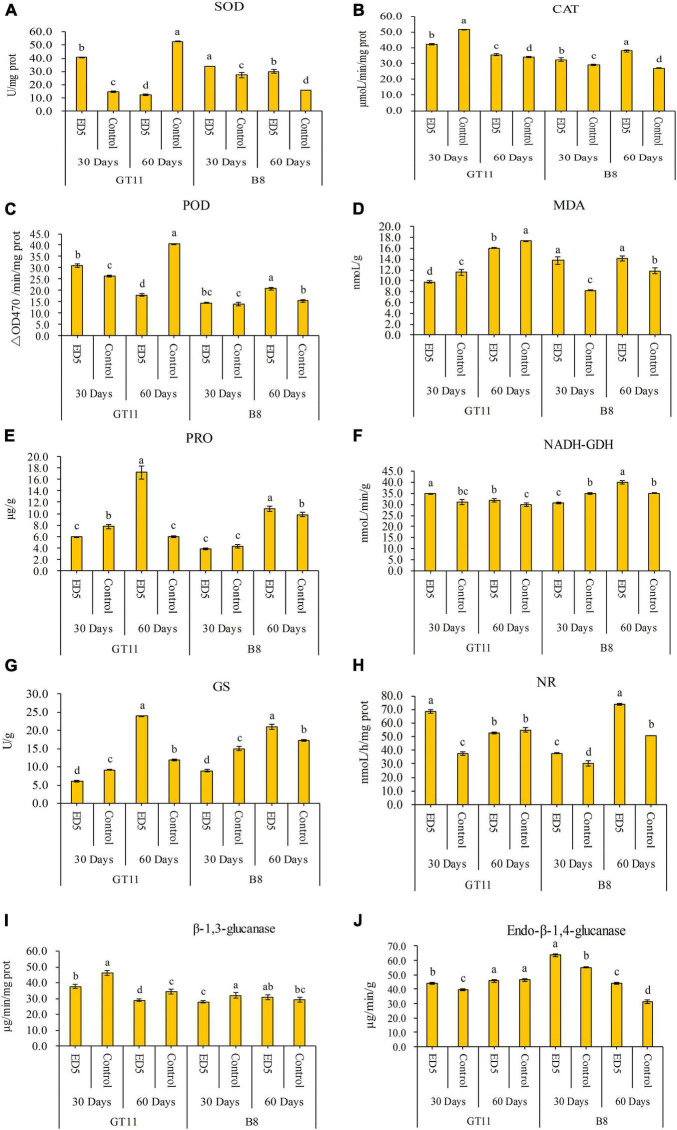
Variations in biochemical parameters in the leaf of sugarcane varieties GT11 and B8 inoculated with *E. roggenkampii* ED5 as compared to control. **(A)** Superoxide dismutase (SOD) activity, **(B)** catalase (CAT) activity, **(C)** peroxidase (POD) activity, **(D)** malondialdehyde (MDA) content, **(E)** proline (Pro) content, **(F)** NADH-glutamate dehydrogenase (NADH-GDH) activity, **(G)** glutamine synthetase (GS) activity, **(H)** nitrate reductase (NR) activity, **(I)** β-1,3-glucanase (β-1,3-GA) activity, and **(J)** endo-β-1,4-glucanase activity. Plant samples were collected at 30 and 60 days after the inoculation. At the level of *p* < 0.05, different letters indicate significant changes between treatments.

The results of biochemical analyses are shown in Figure 1. The SOD activity in sugarcane variety GT11 was enhanced at 30 days and decreased at 60 days after inoculation with ED5 as compared to control; however, it was higher in sugarcane variety B8 after the inoculation of strain ED5 at both time points (30 and 60 days) (Figure 1A). The CAT activity was found higher in both varieties inoculated with ED5 as relative to control, except for GT11 at 30 days (Figure 1B). The POD activity was found higher in GT11 and did not change in B8 at 30 days, whereas it was reduced in GT11 and increased in B8 at 60 days (Figure 1C). The MDA content was lower in GT11 and higher in B8 at specific time points as compared to control (Figure 1D). The proline content was initially decreased (30 days) and then increased (60 days) in GT11, whereas it initially did not change significantly and then it was higher at 60 days in B8 as compared to control (Figure 1E).

The NADH-GDH activity was higher at both time points in GT11 and lower at 30 days and higher at 60 days in B8 (Figure 1F). The GS activity was observed lower and then higher in both varieties at 30 and 60 days after inoculation with strain ED5 (Figure 1G). The NR activity showed different patterns with different sugarcane varieties, which was initially increased and then did not change significantly in GT11, but higher in B8 at both periods (Figure 1H).

The β-1,3-glucanase activity was lower in GT11 at each time point and did not change in B8 as compared to control (Figure 1I). The endo-β-1,4-glucanase activity in B8 was higher at both time points, and that in GT11 it was found higher at 30 days and did not change 60 days after inoculation with ED5 (Figure 1J).

### Contents of Phytohormone

**FIGURE 2:**
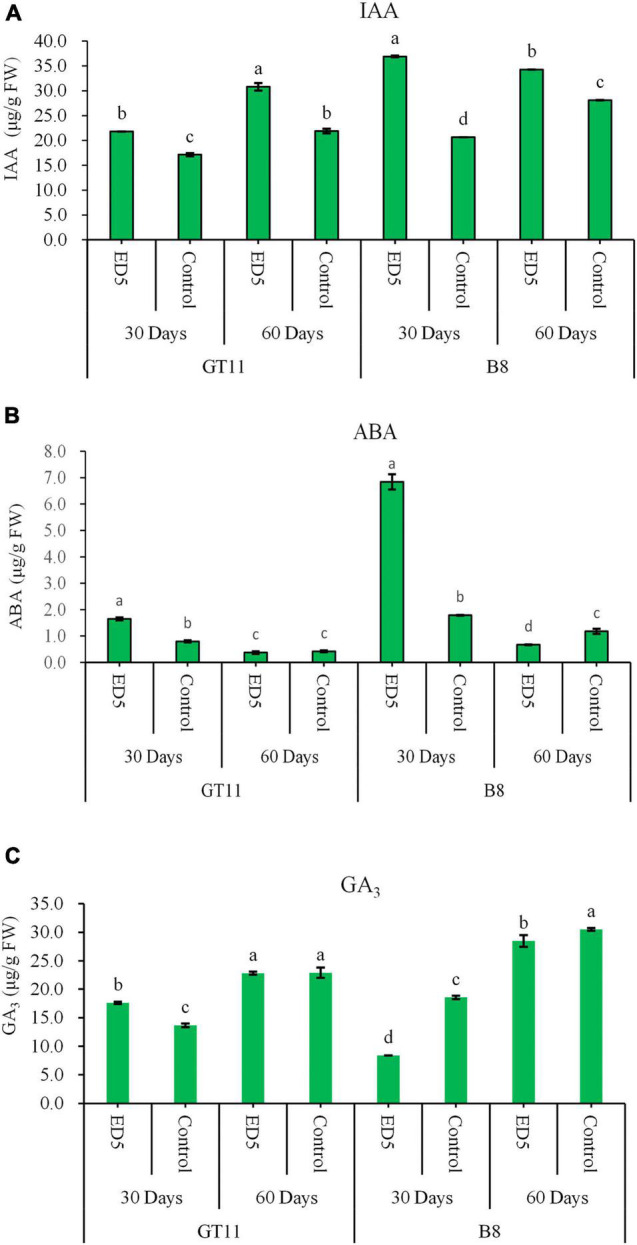
Variations in contents of phytohormones in the leaf of sugarcane varieties GT11 and B8 inoculated with *E. roggenkampii* ED5 as compared to control at 30 and 60 days after the treatment. **(A)** Indole acetic acid (IAA), **(B)** abscisic acid (ABA), and **(C)** gibberellin (GA_3_). At the level of *p* < 0.05, different letters indicate significant changes between treatments.

The contents of phytohormones, such as IAA, GA_3_, and ABA, were measured in both sugarcane varieties GT11 and B8 at 30 and 60 days after inoculation with *E. roggenkampii* ED5. The IAA content was higher in both varieties at 30 and 60 days after the inoculation as compared to control (Figure 2A). The ABA content was increased in both varieties at 30 days, but did not change in GT11 at 60 days and was reduced in B8 at 60 days (Figure 2B). For GA_3_, the content was increased in GT11 and decreased in B8 at 30 days after the inoculation, but did not change significantly in GT11 and was decreased in B8 at 60 days (Figure 2C).

### Analysis of Nitrogen Utilization in Sugarcane

**FIGURE 3:**
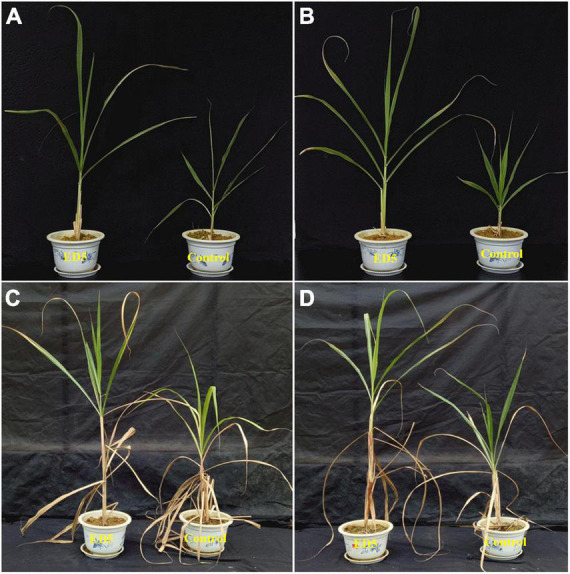
Sugarcane growth promotion by inoculation of *E. roggenkampii* ED5 as compared to control. **(A)** GT11 (30 days), **(B)** B8 (30 days), **(C)** GT11 (60 days), and **(D)** B8 (60 days).

The percentage of ^15^N and total nitrogen was also estimated in both sugarcane varieties at 60 days post-inoculation of ED5 (Table 1). The percentage of ^15^N in the root tissues of both sugarcane varieties was significantly higher in the treatment inoculated with ED5 than control. In leaf tissues, it was higher in B8 and did not change significantly in GT11 (*p* < 0.05; Table 1). However, both sugarcane varieties GT11 and B8 had significantly higher total nitrogen concentration in leaf and root of sugarcane inoculated with ED5 than control (*p* < 0.05; Table 1). The growth of both sugarcane varieties was increased after the inoculation of strain *E. roggenkampii* ED5 as compared to control (Figure 3).

**TABLE 1:** Effect of strain ED5 on percentage of ^15^N and total nitrogen content in two sugarcane varieties.

Sample		^15^N atom%		Total N%	
		Root	Leaf	Root	Leaf
GT11	ED5	1.023 ± 0.088^ab^	1.061 ± 0.003^b^	0.711 ± 0.019^a^	1.257 ± 0.097^ab^
	C	0.923 ± 0.044^c^	1.042 ± 0.008^b^	0.539 ± 0.007^b^	1.115 ± 0.038^bc^
B8	ED5	1.100 ± 0.020^a^	1.268 ± 0.102^a^	0.715 ± 0.061^a^	1.340 ± 0.094^a^
	C	0.922 ± 0.066^d^	0.933 ± 0.046^c^	0.406 ± 0.009^c^	1.032 ± 0.057^c^

*All data points are presented as mean ± SE (*n* = 3). Different lowercase letters indicate a significant difference at *p* < 0.05.*

### Quantitative Identification of Sugarcane Leaf Protein Using Tandem Mass Tags

Tandem Mass Tags labeling was used to identify the DEPs in GT11 and B8 varieties inoculated with *E. roggenkampii* ED5 as relative to control. A sum of 27,508 proteins from 73,823 peptides, matching 301,280 spectrograms, was observed (Supplementary Table 1). The distribution of peptide matching error, peptide number, peptide length, and protein molecular is shown in Supplementary Figures 1A–D, respectively. The percentage of protein sequences with coverage of (0, 1%) was 2.83% and the coverage of (1, 5%) and (40, 60%) was 15.4%. The percentage of protein sequences with the coverage of (5, 10%), (10, 20%), (20, 40%), and (60, 80%) was 14.17, 20.04, 26.23, and 5.41%, respectively, whereas the protein sequences with coverage more than 80% accounted for 0.52% (Supplementary Figure 2). The heat map was used for correlation analysis of different duplicate samples and the data showed that the duplicate samples were clustered together (Supplementary Figure 3). The mass spectrometry proteomics data were deposited to the ProteomeXchange Consortium^2^ *via* the iProX partner repository with the data set identifier PXD026390 (Ma et al., 2019).

**FIGURE 4:**
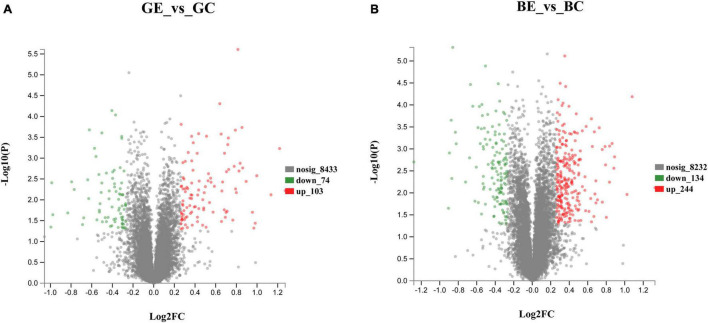
Volcano plots showing the differentially expressed protein allocation in the sugarcane varieties GT11 **(A)** and B8 **(B)** after the inoculation of *E. roggenkampii* ED5 as compared to control. Each data point represented the differential expression of the protein in the volcano plot. Downregulated differentially expressed proteins were shown with green dots, upregulated differentially expressed proteins were marked with red dots, and non-differentially expressed proteins were labeled by gray dots. Protein ratios with *p*-value < 0.05, fold change > 1.20 (upregulated), or < 0.83 (downregulated) were significantly differently expressed.

**FIGURE 5:**
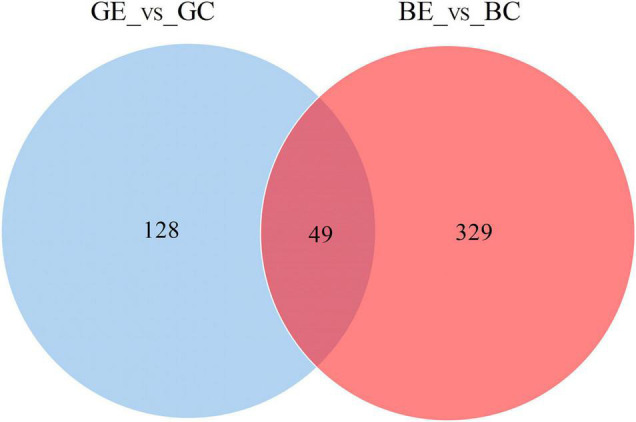
Venn diagrams showing the differentially expressed protein number allocation among the high-efficiency nitrogen-fixing variety B8 and low-efficiency nitrogen-fixing variety GT11 of sugarcane after the inoculation of *E. roggenkampii* ED5 as compared to control.

When compared to control, the DEPs were identified by using a fold-change threshold of > 1.20 or < 0.83 (*p* < 0.05). We analyzed the DEPs in both sugarcane varieties GT11 and B8 between the treatments inoculated with *E. roggenkampii* ED5 (GE, BE) and controls (GC, BC). A total of 177 DEPs, 103 upregulated and 74 downregulated (74), were marked in GT11 between GC and GE (Figure 4A). In contrast, a total of 378 DEPs, 244 upregulated and 134 downregulated, were identified in B8 between BC and BE (Figure 4B). A Venn diagram was constructed to show the common and different DEP numbers between GT11 and B8 varieties (Figure 5). Annotation of the DEPs in the GO, COG, and KEGG biological databases obtained the relevant annotation information (Supplementary Figures 4–6). The key DEPs were related to the growth of plant and mainly involved in the biological functions, such as nitrogen metabolism, photosynthesis, response to oxidative stress, starch and sucrose metabolism, hydrolase activity, oxidative phosphorylation, glutathione metabolism, phenylpropanoid metabolic process, response to stress, and proline catabolic process (Table 2).

**TABLE 2:** List of differentially expressed proteins in two sugarcane varieties inoculated with the diazotroph *E. roggenkampii* ED5 as compared to control.

Biological function	Protein ID	Protein name	GE_VS_GC		BE_VS_BC	
			Fold change	*p*- value	Fold-change	*p*- value
Nitrogen metabolism	TRINITY_DN2556_c0_g1_i16_orf1	Uncharacterized	0.9489	0.5063	1.2144	0.0016
	TRINITY_DN36336_c0_g1_i1_orf1	Bark storage protein A	1.2238	0.1560	1.3781	0.0004
	TRINITY_DN2872_c1_g2_i3_orf1	Glutamine synthetase	0.9167	0.2719	0.7887	0.0178
Photosynthesis	TRINITY_DN4494_c0_g1_i9_orf1	Photosystem II 10 kDa polypeptide, chloroplastic	1.1514	0.2035	0.7765	0.0079
	TRINITY_DN1905_c0_g1_i7_orf1	Chlorophyll a-b binding protein	1.2161	0.0013	0.8413	0.0002
	TRINITY_DN28845_c0_g1_i1_orf1	Chlorophyll a-b binding protein M9	1.2795	0.0212	0.9462	0.1416
	TRINITY_DN9512_c0_g1_i1_orf1	Light-harvesting chlorophyll a/b binding protein 1	1.3122	0.0148	0.8494	0.0087
	TRINITY_DN4271_c0_g1_i5_orf1	Photosystem I reaction center subunit N	0.9589	0.3685	0.7929	0.0044
	TRINITY_DN5666_c0_g1_i8_orf1	Ferritin-1, chloroplastic	0.9637	0.4327	1.2141	0.0442
	TRINITY_DN21412_c0_g1_i2_orf1	Magnesium-chelatase subunit Chl I, chloroplastic	0.9245	0.0530	0.8155	0.0022
	TRINITY_DN7180_c1_g1_i1_orf1	Phosphoenolpyruvate carboxylase 2	1.0317	0.2964	1.2020	0.0012
	TRINITY_DN14316_c0_g1_i2_orf1	Ribulose-phosphate 3-epimerase	0.9805	0.7011	1.2317	0.0007
	TRINITY_DN11044_c0_g1_i5_orf1	Fructose-bisphosphate aldolase	0.8610	0.0072	1.2207	0.0005
Response to oxidative stress	TRINITY_DN13997_c0_g1_i5_orf1	Peroxidase 52 precursor	1.2608	0.0007	1.0613	0.2275
	TRINITY_DN6509_c0_g1_i4_orf1	Peroxidase 2	1.7162	0.0016	1.1063	0.2569
	TRINITY_DN10001_c0_g1_i1_orf1	Peroxidase 2	1.4144	0.0048	1.1750	0.0701
	TRINITY_DN39002_c0_g1_i4_orf1	Pathogenesis-related protein 10	1.5022	0.0047	0.9508	0.2615
	TRINITY_DN66958_c0_g1_i5_orf1	Actin, gamma	1.6364	0.0188	0.9434	0.3663
	TRINITY_DN9680_c0_g1_i7_orf1	Peroxidase 5	1.2741	0.0138	1.1025	0.2839
	TRINITY_DN36448_c2_g1_i1_orf1	Peroxidase 70	1.4034	0.0067	1.3055	0.0591
	TRINITY_DN27318_c0_g2_i1_orf1	Peroxidase 2	1.7856	0.0013	1.2005	0.2336
	TRINITY_DN9788_c0_g2_i2_orf1	Peroxidase 1	0.9790	0.6559	1.2542	0.0222
	TRINITY_DN29188_c0_g1_i1_orf1	Peroxidase 4	1.1414	0.0854	1.2022	0.0261
	TRINITY_DN18298_c0_g1_i4_orf1	Chloroplastic isoform X2	1.0719	0.3912	0.8015	0.0151
	TRINITY_DN7490_c0_g1_i2_orf1	Peroxidase 1	1.0569	0.1173	0.8141	0.0046
	TRINITY_DN9680_c0_g2_i2_orf1	Peroxidase 5	1.1313	0.0496	1.2314	0.0130
	TRINITY_DN989_c0_g1_i4_orf1	Chloroplastic lipocalin isoform X1	1.0540	0.8166	0.7801	0.0101
	TRINITY_DN5152_c0_g3_i1_orf1	Peroxidase 17	1.0625	0.1953	0.6942	0.0010
	TRINITY_DN5152_c0_g1_i1_orf1	Peroxidase 47	1.0802	0.0519	0.7926	0.0027
	TRINITY_DN7713_c0_g1_i4_orf1	Peptide methionine sulfoxide reductase A2-1	0.9019	0.1053	1.2071	0.0009
	TRINITY_DN4778_c0_g2_i1_orf1	Peroxidase 5	1.0970	0.0894	1.3257	0.0191
	TRINITY_DN13406_c0_g1_i4_orf1	Putative L-ascorbate peroxidase 6	1.0528	0.1103	0.8202	0.0016
	TRINITY_DN30136_c0_g1_i1_orf1	Peroxidase 51 isoform X4	1.1106	0.2583	1.2415	0.0151
	TRINITY_DN28132_c0_g1_i6_orf1	Positive regulation of response to oxidative stress	1.1312	0.0260	0.6883	0.0002
	TRINITY_DN87423_c0_g1_i2_orf1	Lactoylglutathione lyase	0.9457	0.2403	1.3497	0.0258
	TRINITY_DN1706_c0_g2_i3_orf1	Temperature-induced lipocalin	0.8902	0.0065	1.3167	0.0001
Starch and sucrose metabolism	TRINITY_DN2350_c0_g1_i5_orf1	Beta-glucosidase	1.2372	0.0399	0.8372	0.0300
	TRINITY_DN64709_c0_g1_i8_orf1	Granule-bound starch synthase 1b	0.7280	0.0023	1.0807	0.0318
	TRINITY_DN9378_c0_g1_i10_orf1	Beta-fructofuranosidase 1	0.9605	0.4301	1.2156	0.0027
	TRINITY_DN34918_c0_g1_i4_orf1	Endoglucanase 10	1.0680	0.3176	1.3662	0.0019
	TRINITY_DN3827_c0_g2_i1_orf1	Soluble starch synthase 2-3	0.9315	0.0566	1.2804	0.0023
	TRINITY_DN6813_c0_g1_i2_orf1	Hypothetical protein VIR_4G276200v2	0.9641	0.5914	1.3861	0.0089
Hydrolase activity	TRINITY_DN36731_c0_g1_i4_orf1	Chitinase 1	1.3900	0.0099	0.8335	0.0141
	TRINITY_DN134_c0_g1_i6_orf1	Chitinase 6	1.2903	0.0040	0.9598	0.4741
	TRINITY_DN6585_c0_g1_i5_orf1	Glycosyl hydrolases family 18	1.2174	0.0106	1.1291	0.2829
	TRINITY_DN18844_c0_g1_i1_orf1	Xylanase inhibitor protein 1	1.1573	0.0804	0.6932	0.0042
	TRINITY_DN49939_c0_g1_i1_orf1	Chitinase-B, partial	1.1976	0.0028	0.6828	0.0003
	TRINITY_DN7175_c0_g1_i4_orf1	Chitinase 4	1.1876	0.1116	0.6250	0.0016
	TRINITY_DN57445_c0_g1_i1_orf1	Chitinase 12	1.0049	0.9403	1.6310	0.0144
	TRINITY_DN23796_c0_g1_i1_orf1	Chitinase 7	0.9721	0.6964	1.3207	0.0018
	TRINITY_DN11524_c0_g1_i1_orf1	Chitinase 3	1.4395	0.0035	1.0552	0.4830
	TRINITY_DN48283_c0_g1_i3_orf1	β-1,3-Glucanase A	1.9970	0.0026	0.9018	0.2496
	TRINITY_DN13965_c0_g3_i3_orf1	Xyloglucan endotransglycosylase homolog 1	1.3531	0.0162	1.0301	0.6392
	TRINITY_DN44978_c0_g2_i1_orf1	Endoglucanase 24	1.0744	0.2242	1.3088	0.0464
	TRINITY_DN44833_c0_g3_i1_orf1	Glucan endo-1,3-β-glucosidase 8	1.1091	0.1869	0.7765	0.0020
	TRINITY_DN12673_c0_g1_i2_orf1	Beta-amylase	0.8576	0.0426	1.3799	0.0014
	TRINITY_DN13379_c0_g2_i1_orf1	Xyloglucan endotransglycosylase/hydrolase protein 8	1.1499	0.1426	1.3217	0.0262
	TRINITY_DN2190_c1_g1_i1_orf1	Glucan endo-1,3-β-glucosidase 8	1.1828	0.0131	0.7402	0.0046
Oxidative phosphorylation	TRINITY_DN11887_c0_g1_i4_orf1	Xanthine dehydrogenase	1.2288	0.0300	0.9612	0.3997
	TRINITY_DN11186_c1_g1_i1_orf1	Vacuolar H^+^-pyrophosphatase	1.1593	0.0419	0.8170	0.0345
	TRINITY_DN81886_c0_g3_i1_orf1	Cytochrome c oxidase subunit 6b-3	0.9920	0.8964	1.2189	0.0019
Glutathione metabolism	TRINITY_DN8335_c0_g1_i6_orf1	Protein IN2-1 homolog B	0.8117	0.0077	1.0626	0.0654
	TRINITY_DN1847_c0_g2_i2_orf1	Probable glutathione S-transferase	1.2904	0.0012	1.0486	0.1696
	TRINITY_DN52066_c0_g1_i1_orf1	6-Phosphogluconate dehydrogenase	2.4157	0.0060	0.9260	0.3145
	TRINITY_DN7939_c0_g1_i3_orf1	Glutathione transferase activity	1.1063	0.2304	0.7815	0.0022
	TRINITY_DN7913_c0_g1_i5_orf1	Glutathione transferase GST 23	0.9933	0.9143	1.2198	0.0455
	TRINITY_DN7939_c0_g1_i12_orf1	Glutathione transferase activity	1.1155	0.1860	0.7442	0.0009
	TRINITY_DN59556_c0_g1_i1_orf1	Glutathione binding	1.0698	0.0809	0.8247	0.0021
	TRINITY_DN7303_c0_g1_i1_orf1	Glutathione transferase 6	1.0752	0.2877	1.2916	0.0060
Phenylpropanoid metabolic process	TRINITY_DN2764_c0_g1_i7_orf1	Phenylalanine ammonia-lyase	1.0518	0.4075	1.3531	0.0014
	TRINITY_DN7445_c0_g1_i1_orf1	Phenylalanine ammonia-lyase	1.0143	0.6097	1.7428	0.0360
	TRINITY_DN3554_c0_g2_i9_orf1	Caffeic acid 3-*O*-methyltransferase	0.9698	0.5382	1.4553	0.0004
	TRINITY_DN25053_c0_g1_i6_orf1	RecName: Full = Probable cinnamyl alcohol dehydrogenase	0.9749	0.5661	1.4237	0.0017
	TRINITY_DN6377_c0_g1_i2_orf1	Cinnamate 4-hydroxylase 2	0.9790	0.5925	1.2328	0.0009
	TRINITY_DN3258_c0_g1_i5_orf1	Caffeoyl-CoA *O*-methyltransferase 1	0.9948	0.9411	1.4156	0.0039
	TRINITY_DN2764_c0_g1_i5_orf1	Phenylalanine ammonia-lyase 3	1.0315	0.6259	1.6522	0.0003
	TRINITY_DN2764_c0_g1_i2_orf1	Phenylalanine ammonia-lyase	1.1193	0.1907	1.8437	0.0028
	TRINITY_DN7445_c0_g2_i1_orf1	Phenylalanine ammonia-lyase	1.1053	0.3301	1.7319	0.0023
	TRINITY_DN10211_c0_g2_i1_orf1	Phenylalanine ammonia-lyase	1.0633	0.4860	1.5690	0.0009
Response to stress	TRINITY_DN54500_c0_g1_i1_orf1	Aquaporin PIP2-4	1.2925	0.0077	0.8774	0.0653
	TRINITY_DN14281_c0_g1_i1_orf1	Aquaporin	1.2247	0.0182	0.9078	0.0720
	TRINITY_DN99555_c0_g1_i1_orf1	Zeamatin	1.6498	0.0003	0.8677	0.2602
	TRINITY_DN3871_c1_g1_i2_orf1	Aquaporin	1.2061	0.0292	0.8488	0.0137
	TRINITY_DN4325_c0_g1_i10_orf1	Putative tubulin alpha-3 chain	0.7691	0.0049	1.0671	0.2141
	TRINITY_DN23009_c0_g4_i1_orf1	Aquaporin PIP2-5	1.3518	0.0058	0.9510	0.2784
	TRINITY_DN23009_c0_g2_i1_orf1	Aquaporin PIP2-4	1.2839	0.0076	1.0108	0.8198
	TRINITY_DN3871_c1_g1_i6_orf1	Aquaporin	1.2250	0.0032	0.9656	0.4947
	TRINITY_DN5728_c0_g1_i5_orf1	Hypothetical protein Zm00014a_016292	0.6923	0.0025	0.9491	0.4470
	TRINITY_DN6371_c0_g2_i1_orf1	Probable mediator of RNA polymerase II transcription subunit 37c	0.8271	0.0134	1.0918	0.0114
	TRINITY_DN14_c0_g2_i5_orf1	Hypothetical protein SORBI_3009G009600	1.7319	0.0055	1.0355	0.6792
	TRINITY_DN52792_c0_g1_i3_orf1	Linoleate 9S-lipoxygenase1	1.6253	0.0200	1.4298	0.0535
	TRINITY_DN1151_c4_g1_i4_orf1	Heat shock 70 kDa protein 17	0.7688	0.0041	0.9823	0.7579
	TRINITY_DN13126_c0_g1_i10_orf1	Endoplasmin-like protein	0.8014	0.0075	0.9723	0.3196
	TRINITY_DN28994_c0_g1_i5_orf1	Hypothetical protein SORBI_3001G158400	0.8129	0.0475	1.1928	0.0139
	TRINITY_DN5845_c0_g2_i1_orf1	Copper transport protein ATX1 isoform X1	1.0291	0.6350	0.5472	0.0047
	TRINITY_DN11930_c0_g1_i2_orf1	Annexin D4	0.9556	0.2712	1.2460	0.0076
	TRINITY_DN17619_c0_g1_i11_orf1	Uncharacterized protein LOC8056876	0.8693	0.4870	1.3447	0.0056
	TRINITY_DN3871_c0_g1_i7_orf1	Probable aquaporin PIP2-7	0.9806	0.5562	1.2927	0.0076
	TRINITY_DN3546_c0_g1_i2_orf1	Glutamine synthetase, chloroplastic	1.0625	0.3098	1.2543	0.0003
	TRINITY_DN23009_c0_g3_i1_orf1	Aquaporin PIP2-6	1.0791	0.0001	0.8087	0.0176
	TRINITY_DN2562_c0_g1_i4_orf1	Hypothetical protein SORBI_3009G009400	1.0043	0.9525	1.5501	0.0134
	TRINITY_DN28232_c0_g1_i1_orf1	Uncharacterized protein LOC100272711 precursor	0.9782	0.6329	1.2121	0.0123
	TRINITY_DN2562_c0_g1_i7_orf1	Subtilisin-chymotrypsin inhibitor-2B	1.0695	0.3947	1.3053	0.0074
	TRINITY_DN14_c0_g2_i1_orf1	Subtilisin-chymotrypsin inhibitor-2B	1.0695	0.3947	1.3053	0.0074
	TRINITY_DN24351_c0_g1_i1_orf1	Annexin p35	0.9046	0.0287	1.2125	0.0038
	TRINITY_DN17376_c0_g1_i1_orf1	Hypothetical protein SORBI_3009G009700	1.0996	0.3118	1.4561	0.0072
	TRINITY_DN29723_c0_g1_i4_orf1	Osmotin-like protein	1.0860	0.3307	1.5584	0.0002
	TRINITY_DN3987_c0_g1_i16_orf1	Isoflavone reductase homolog IRL	0.9646	0.2749	1.2788	0.0071
	TRINITY_DN54500_c0_g2_i1_orf1	Aquaporin PIP1-5	1.1087	0.1842	0.5622	0.0004
	TRINITY_DN2562_c0_g1_i1_orf1	Subtilisin-chymotrypsin inhibitor-2B	1.0518	0.1232	1.3668	0.0094
Proline catabolic process	TRINITY_DN16632_c0_g1_i2_orf1	Proline dehydrogenase 2, mitochondrial	0.9659	0.3997	1.2036	0.0004
	TRINITY_DN3173_c0_g1_i4_orf1	Proline dehydrogenase 2, mitochondrial	0.9131	0.0572	1.2762	0.0000

*GE and GC represented the treatment inoculated with *E. roggenkampii* ED5 and control inoculated with water for G11; BE and BC represented the treatment inoculated with *E. roggenkampii* ED5 and control inoculated with water for B8. Differentially expressed proteins: fold-change 1.20 (upregulated) or < 0.83 (downregulated), and *p*-value < 0.05.*

### Gene Ontology Analysis of Differentially Expressed Proteins

**FIGURE 6:**
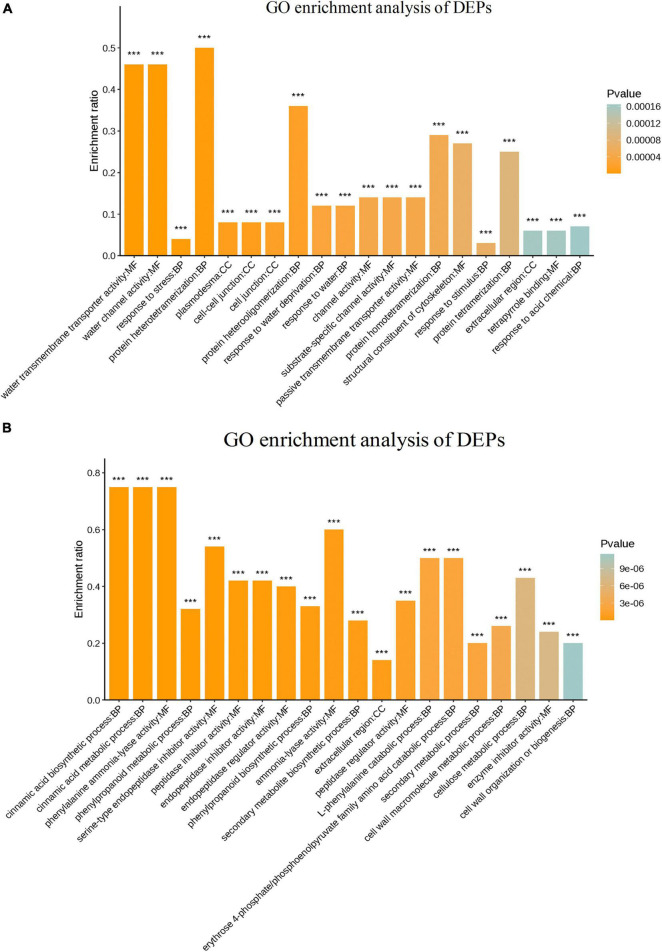
GO enrichment of differentially expressed proteins in **(A)** GT11 and **(B)** B8 varieties of sugarcane inoculated with *E. roggenkampii* ED5 as compared to control. The abscissa represents the GO term and the ordinate represents the enrichment rate. The color gradient of the column represents the significance of enrichment, where *p* or FDR < 0.001 was marked as ***, *p* or FDR < 0.01 was marked as **, and *p* or FDR < 0.05 was marked as *.

The putative functions of DEPs were investigated in the two sugarcane varieties GT11 and B8 inoculated with *E. roggenkampii* ED5 using GO enrichment analysis and classified into the groups related to biological, cellular, and molecular processes (Figure 6). The results of the GO enrichment analysis showed that response to stimulus (GO: 0050896) was the most significantly enriched term under the biological process for GT11. Additionally, the terms response to stress (GO: 0006950), hydrogen peroxide catabolic process (GO: 0042744), photosynthesis (GO: 0009765), response to oxidative stress (GO: 0006979), response to water (GO: 0009415), and response to acid chemical (GO: 0001101) were enriched in GT11 (Figure 6A). The GO term enrichment for B8 is mainly focused on phenylpropanoid metabolic process (GO: 0009698), secondary metabolite biosynthetic process (GO: 0044550), phenylpropanoid biosynthetic process (GO: 0009699), serine-type endopeptidase inhibitor activity (GO: 0004867), peptidase regulator activity (GO: 0061134), and peptidase inhibitor activity (GO: 0030414) (Figure 6B).

### Pathway Analysis of Differentially Expressed Proteins by Kyoto Encyclopedia of Genes and Genomes

**FIGURE 7:**
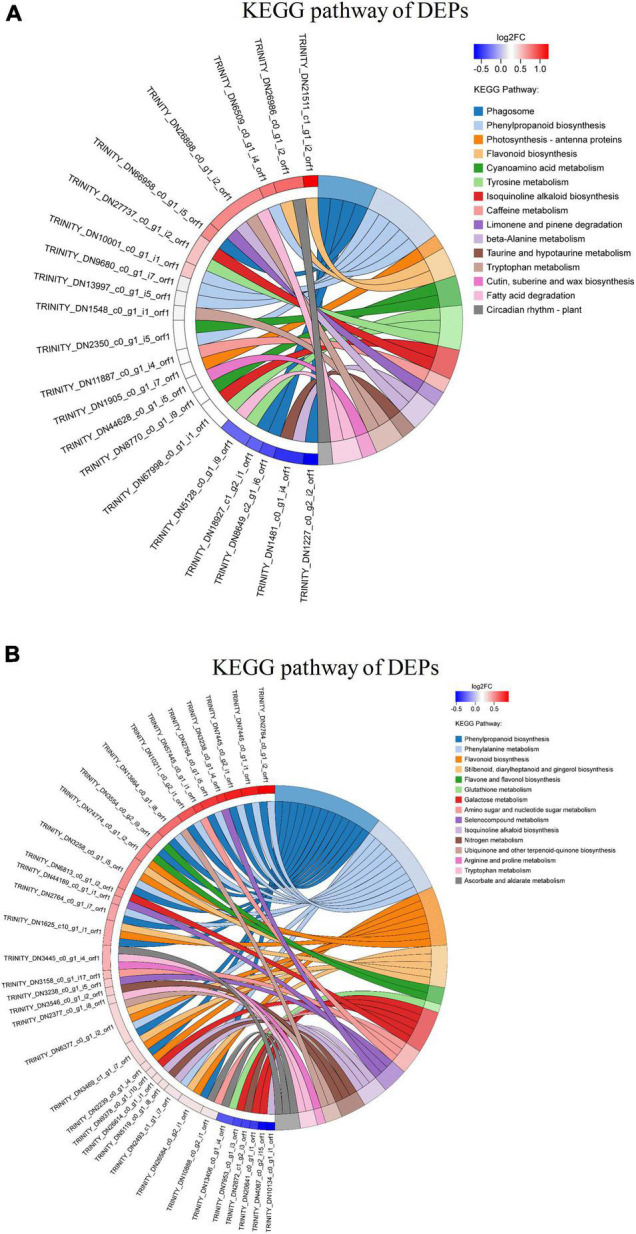
KEGG enrichment of differentially expressed proteins in **(A)** GT11 and **(B)** B8 varieties of sugarcane inoculated with *E. roggenkampii* ED5 as compared to control. The KEGG-enriched string diagram depicted the relationship between the target protein set and the KEGG pathway annotation and enrichment. The left side indicated the proteins and the log2FC was displayed in order from top to bottom. The larger the log2FC, the greater the difference in the expression of the upregulated proteins. The smaller the log2FC, the greater the difference in the expression of the downregulated proteins. The closer the log2FC was to 0, the smaller the difference in the fold of differential expression of the proteins. The right gave the name of the KEGG pathway that enriched the target protein and the Z score. The count represented the number of proteins associated with this pathway. For the total number of target proteins, up represented the number of upregulated proteins linked in this pathway, and down represented the number of downregulated proteins involved in this pathway. Z score > 0 meant that there were more upregulated proteins than downregulated proteins involved in this pathway, and this pathway was more likely to be activated. Z score < 0 indicated that there were fewer upregulated proteins than downregulated proteins involved in this pathway, and this pathway was more likely to be inhibited. In this enrichment chart, the top 50 target proteins with a large number of annotation pathways were selected. The pathways contained these target proteins in the enrichment and the pathways with the enrichment significance *p-*value of these target proteins were ranked in the top 15 for display.

Analysis of the KEGG pathways was done to understand the effective functional information of identified DEPs in sugarcane inoculated with ED5. The 177 DEPs in GT11 were linked to 61 pathways, and 378 DEPs in B8 were mapped to 77 pathways (Supplementary Figure 6). Besides, the function enrichment of DEPs in KEGG pathways was analyzed using Fisher’s exact test. When the corrected *p-*value is < 0.05, the DEP was considered to be significantly enriched. The top 50 target proteins with a large number of annotation pathways and the pathways that contained these target proteins in the enrichment results and the pathways with the significance *p*-value of the top 15 are displayed in the string diagram (Figure 7). The pathways for GT11 were mainly enriched with phenylpropanoid biosynthesis (map00940), photosynthesis-antenna proteins (map00196), beta-alanine metabolism (map00410), tryptophan metabolism (map00380), cutin, suberin, and wax biosynthesis (map00073), fatty acid degradation (map00071), and circadian rhythm plant (map04712) (Figure 7A). The pathways for B8 were mainly enriched with phenylpropanoid biosynthesis (map00940), phenylalanine metabolism (map00360), glutathione metabolism (map00480), galactose metabolism (map00052), amino sugar and nucleotide sugar metabolism (map00520), nitrogen metabolism (map00910), arginine and proline metabolism (map00330), tryptophan metabolism (map00380), and ascorbate and aldarate metabolism (map00053) (Figure 7B).

## Discussion

Plant growth-promoting bacteria are important for the growth of plant, and their use in agricultural production has a lot of advantages (Bhattacharyya and Jha, 2012; Kumari et al., 2019). The mechanism of PGPB in promoting the growth of plant is multifaceted, including direct or indirect mechanisms (Goswami et al., 2016). In this study, the inoculation of PGP N-fixing *E. roggenkampii* ED5 strain isolated from the sugarcane roots enhanced the growth and nitrogen fixation in the two sugarcane varieties GT11 and B8 under greenhouse conditions as compared to control (Figure 3). In addition, the results of biochemical analyses, phytohormone detection, and comparative proteome analysis revealed the physiological, biochemical, and molecular mechanisms of the interaction between *E. roggenkampii* ED5 and sugarcane.

### Changes in Biochemical Parameters

Plant growth-promoting bacteria has positive effects on the enhancement of plant antioxidant enzyme activities and growth enhancement (Islam et al., 2014; Benidire et al., 2021). This study also found that after the inoculation of ED5, stress-related enzyme activities (SOD and CAT) and osmotic regulator Pro content in both the sugarcane cultivars were dramatically increased. In contrast, the results of protein expression analysis showed differential expression of Pro and POD in ED5-inoculated sugarcane varieties. Enhanced expression of proline dehydrogenase 2 was observed in B18, but remained unchanged in GT11. Also, increased expression of peroxidase 2 in GT11 and decreased expression of peroxidase 17 in B8 variety were observed.

In addition, some PGPB can produce endogenous hormones required for the growth of plant, such as auxin, cytokinin, gibberellin, ABA, ethylene, salicylic acid, and jasmonic acid, thereby directly regulating the performance of plant (Tsukanova et al., 2017). In this study, the sugarcane endogenous hormone IAA was found higher in both varieties after the inoculation of strain ED5. It implied that the *E. roggenkampii* ED5 has the potential to promote the growth of sugarcane and was consistent with the plant growth of the two sugarcane varieties in the greenhouse. To stimulate the growth of plant, the ABA plays a crucial role in the adaptive response to abiotic environmental challenges, but it is usually linked to growth inhibition of plants or organs (Leung and Giraudat, 1998; Kuromori et al., 2010). In this study, the ABA content of the two sugarcane varieties was higher at 30 days, but did not change in GT11 and lower in B8 at 60 days, suggesting that the inoculation of *E. roggenkampii* ED5 could improve the environment adaptive ability of sugarcane at early stage of growth by increasing the ABA level but this effect is not necessary after the plants have adopted the environment and begun to grow fast. Based on the results of this study, it seems that the effect of inoculation of *E. roggenkampii* ED5 on gibberellin content in sugarcane is variety-dependent. However, no significant changes were observed in the expression of phytohormone-related proteins in both sugarcane varieties inoculated with ED5.

Nitrogen fixation is one of the growth-promoting factors of some PGPB (Jha and Saraf, 2015). Therefore, enhanced activities of nitrogen metabolism-related enzymes are directly associated with nitrogen fixation by PGPB. In this study, the activities of nitrogen metabolism-related enzymes GS and NADH-GDH in the sugarcane leaves were found to be higher in the treatment inoculated with *E. roggenkampii* ED5 than control, indicating the improvement of the nitrogen metabolism by the treatment inoculated with ED5. The endo-β-1,4-glucanase activity was also found higher in the treatment inoculated with *E. roggenkampii* ED5 than control, which could be connected to the faster growth of sugarcane plants in the treatment inoculated with ED5. The activities of NR and β-1,3-glucanase were sugarcane variety- and growth stage-dependent in this study. We also found increased expression of some proteins was related to hydrolase activity and nitrogen metabolism in both sugarcane varieties after the inoculation of strain ED5.

### Nitrogen Fixation Characteristics

Taking advantage of the nitrogen fixation function of PGPB to improve the efficiency of plant nitrogen utilization is an important way to achieve a sustainable development of crop productivity (Figueiredo et al., 2008; Baset et al., 2010; Matse et al., 2020; Basu et al., 2021). As seen earlier, the strain *E. roggenkampii* ED5 showed high nitrogenase activity (29.60 nmoL C_2_H_4_ mg protein/h) through acetylene reduction assay, indicating its N-fixation potential (Guo et al., 2020). To confirm this hypothesis, the ammonium sulfate-^15^N_2_ isotope label dilution method was applied to analyze the utilization rate of nitrogen in sugarcane plant organs such as root and leaf, and the results showed that the concentration of N content in the sugarcane root and leaf was significantly enhanced after the inoculation of *E. roggenkampii* ED5 in both varieties GT11 and B8 as relative to normal plants. Therefore, the *E. roggenkampii* ED5 can effectively fix nitrogen and supply to host sugarcane plants.

Previously, the PGPB in sugarcane rhizosphere is widely investigated and some potent isolates were obtained (Lamizadeh et al., 2016; Wang Z. et al., 2019; Singh et al., 2020). However, the information using proteomics to analyze the interaction mechanism between PGPB and sugarcane is limited. In this study, we used the TMT labeling method to identify the DEPs of two different N-fixing sugarcane varieties GT11 (needs more nitrogen for growth) and B8 (needs less nitrogen for growth) after the inoculation of the diazotroph strain ED5. The results of KEGG enrichment of DEPs indicated that there were three proteins enriched in the N metabolism pathway (map00910) in B8, but not in GT11. Earlier, some researchers have shown that different sugarcane varieties have different N-fixation abilities (Boddey et al., 1991; de Matos Nogueira et al., 2005). Obviously, the inconsistent enrichment of KEGG N-fixing metabolic pathways in the sugarcane varieties with different N-fixation abilities was caused by the interactions of sugarcane varieties with the diazotroph strain ED5.

### Photosynthesis-Related Proteins

Photosynthesis is the major physiological process related to the growth and development of plant, and the relative growth rate (RGR) was positively correlated with the total biomass, leaf mass, and leaf area expansion in plants (Kruger and Volin, 2006). In this study, photosynthesis-related DEPs, such as photosystem II 10 kDa polypeptide, chlorophyll a-b binding protein, photosystem I reaction center subunit N, phosphoenolpyruvate carboxylase 2, and fructose-bisphosphate aldolase, were detected in the treatment inoculated with *E. roggenkampii* ED5 as compared to control. Chlorophyll *a* and *b* and Chl *a*/*b* involve in the light absorption and energy transfer to the photosystem and the reaction center of charge separation in green plants (Gobets et al., 2001; Horn et al., 2007; Kjaer et al., 2020). The results in this study showed that there was a significant upregulation of photosystem-II-related proteins after inoculation with *E. roggenkampii* ED5, which is beneficial to photosynthesis and plant growth. Meanwhile, other photosynthesis-related enzymes, such as photosystem II 10 kDa polypeptide, light-harvesting chlorophyll *a*/*b*, and magnesium-chelatase subunit Chl I, were also upregulated, which further confirmed our previous findings that *E. roggenkampii* ED5 is an efficient diazotroph PGPB strain (Guo et al., 2020). According to relevant research studies, PGPB was able to affect plant photosynthetic capacity, thereby promoting the growth of plant (Su et al., 2015; Samaniego-Gámez et al., 2016; Mathivanan et al., 2017).

### Starch and Sucrose Metabolism-Related Proteins

Sugarcane is a well-known sugar and bioenergy crop worldwide (Li and Yang, 2015). Breeding high sucrose sugarcane varieties is the main research purpose of breeders (Jackson, 2005). In this experiment, differential expression of proteins related to sucrose metabolism, such as beta-glucosidase, granule-bound starch synthase 1b, beta-fructofuranosidase 1, endoglucanase 10, and soluble starch synthase 2-3, was identified after the inoculation of ED5 in sugarcane. Among them, the protein soluble starch synthase 2-3 (ID: TRINITY_DN3827_c0_g2_i1_orf1) was upregulated with fold-change of 1.237 (*p*-value = 0.002) in B8 (Table 2). Soluble starch synthase (SSs) is mainly associated with the synthesis of amylopectin (Cao et al., 1999; Myers et al., 2000), including SSI, SSII, SSIII, and SSIV subtypes (Kossmann et al., 1999). SSII and SSIII usually participate in the extension of short chains (Edwards et al., 1999; Delvallé et al., 2005). Moreover, SSs can transfer the glucose residues of adenosine diphosphate glucose (ADPG) to the non-reducing end of the glucan chain through α-1,4-glycosidic bonds and play a major role in starch and sucrose metabolisms (Komor, 2000; Xue et al., 2019). These results implied that *E. roggenkampii* ED5 might contribute to enhance the activity of sucrose synthesis in sugarcane.

### Stress-Related Proteins

Plant growth-promoting bacteria enhances the resistance of plants to stress, thereby indirectly promoting the growth of plant. Some studies have shown that PGPB can effectively enhance the response of plants to stresses, such as water-deficit conditions (Ngumbi and Kloepper, 2016), salinity (Raklami et al., 2019), pathogens (Siddiqui, 2005), and heat and heavy metals (Raklami et al., 2019). Our previous research also predicted that the *E. roggenkampii* ED5 genome had some vital abiotic stress-related genes, such as those encoding cold and heat shock proteins (HSPs), heavy metal, and drought resistance proteins, and we speculated that the strain ED5 has the potential to resist abiotic stress (Guo et al., 2020). In this study, the GO enrichment of DEPs was focused on several abiotic stress-related proteins, including aquaporin PIP2-4 (protein ID: TRINITY_DN54500_c0_g1_i1_orf1), heat shock 70 kDa protein 17 (protein ID: TRINITY_ DN1151 _c4_g1_i4_orf1), copper transport protein ATX1 isoform X1 (protein ID: TRINITY_DN5845 _c0 _g2 _i1_orf1), GS and chloroplastic (protein ID: TRINITY_DN3546_c0_g1_i2_orf1), and osmotin-like protein (protein ID: TRINITY_DN29723_c0_g1_i4_orf1). These results further confirmed that the tolerance of sugarcane to abiotic stress may be improved after the inoculation of *E. roggenkampii* ED5. Recently, proteomic approaches have been widely used in revealing the molecular mechanism of resistance of plant to abiotic stress, especially in salt and drought tolerance in crops under field conditions (Gautam et al., 2020). In this study, some DEPs related to abiotic stress were observed with TMT proteomics, which provides a reference for further exploring the molecular mechanisms of the interaction between the strain ED5 and sugarcane to improve resistance of sugarcane to abiotic stress.

In addition, GO enrichment analysis showed some DEPs involving in glutathione metabolism pathway, such as glutathione *S*-transferase (protein ID: TRINITY_DN1847_c0_g2_i2_orf1), 6-phosphogluconate dehydrogenase (protein ID: TRINITY_DN52066_c0_g1_i1_orf1, glutathione transferase (TRINITY_DN7939_c0_g1_i3_orf1), glutathione transferase GST 23 (protein ID: TRINI TY_DN7913_c0_g1_i5_orf1), glutathione binding (protein ID: TRINITY_DN59556_c0_g1_i1_orf1), and glutathione transferase 6 (protein ID: TRINITY_DN7303_c0_g1_i1_orf1), were detected after the inoculation of ED5. The glutathione plays a significant role in the maintenance of antioxidant ability of plant tissues and regulation of redox-sensitive signal transduction (Sen, 1999; Cnubben et al., 2001; Hérouart et al., 2002). The level of glutathione was closely related to the tolerance of plants to various biological heterologous substances and environmental stresses (May et al., 1998; Nianiou-Obeidat et al., 2017). In this study, some glutathione metabolism-related DEPs were identified, which were related to the improvement of tolerance of plant to various stresses, the enhancement of plant defense system, and the enhanced plant growth in sugarcane after the inoculation of ED5. Peroxidase is a key enzyme responsible for the synthesis of resistance inducers (Perkins-Veazie et al., 1996). In this study, the significant enrichment of peroxidase was observed in sugarcane after inoculation with strain ED5.

Moreover, in this study, the phenylpropanoid metabolic process of DEPs was also enriched by the KEGG and GO databases. Recent studies have shown that plants respond to environmental stresses during growth and several factors participate in the metabolism of propanol and regulation of the homeostasis of phenylpropanol (Dong and Lin, 2021). The phenylpropanoid metabolic process also contributes to abiotic and biotic stresses (Nakabayashi and Saito, 2015; Sharma et al., 2019). Some phenylalanine ammonia-lyases were enriched in GO analysis. The most fold-change of GO term in phenylpropanoid metabolic was phenylalanine ammonia-lyase (protein ID: TRINITY_DN27 64_c0 _g1_i2_orf1), 1.844 (*p*-value = 0.003). Our previous study also found the presence of key genes related to abiotic stress resistance in the ED5 genome (Guo et al., 2020). Similarly, proteomics analysis revealed several vital stress-related DEPs, including enrichment of phenylpropanoid metabolic proteins in the GO and KEGG databases. All these results implied that sugarcane may enhance resistance and strengthen self-defense ability through various metabolic pathways after inoculation with *E. roggenkampii* ED5.

### Hydrolase-Related Proteins

Some key hydrolases, such as chitinase, glycosyl hydrolases, and glucan endo-1,3-β-glucosidase, play a major role in resistance of plant to biotic or abiotic stresses (Flach et al., 1992; Beffa et al., 1993; Gaudioso-Pedraza and Benitez-Alfonso, 2014). Previously, we found that the *E. roggenkampii* ED5 secreted hydrolases such as cellulase, chitinase, endoglucanase, and protease, and the related genes were also predicted in its genome (Guo et al., 2020). In this study, the GO enrichment of DEPs displayed some hydrolases, with the chitinases being the most enriched hydrolases, including chitinase 1, chitinase 3, chitinase 4, chitinase 6, and chitinase 12. Chitinase plays a crucial role in plant defense, particularly against diseases (Sharma et al., 2011; Kumar et al., 2018; Liu et al., 2020). The strain ED5 has a high inhibitory effect on five kinds of pathogenic fungi *in vitro* plate confrontation test, and several biocontrol-related coding genes were predicted in its genome (Guo et al., 2020). In this study, higher enrichment of the proteins related to hydrolases confirmed our previous hypothesis that the TMT proteomics study can further verify the biocontrol potential of the strain ED5. This isolate may have value in the application of sugarcane biocontrol in future.

## Conclusion

In this study, TMT labeling and LC-MS/MS analyses were carried out to study the comparative protein profiles of two different sugarcane varieties GT11 and B8 inoculated with *E. roggenkampii* ED5. Some key proteins, such as those involved in nitrogen metabolism, photosynthesis, starch and sucrose metabolism, response to oxidative stress, hydrolysis, oxidative phosphorylation, glutathione metabolism, phenylpropanoid metabolic process, and response to stresses, were enriched in the GO and KEGG databases. Furthermore, the research revealed that *E. roggenkampii* ED5 has positive effects on improving nitrogen utilization in sugarcane and promoting plant growth in sugarcane. Increases in the contents of IAA and proline and activities of oxidation defensive enzymes and nitrogen metabolism enzymes were found after inoculated with strain ED5. Therefore, this strain may have the potential to improve sugarcane productivity and eco-environmental sustainability.

## Data Availability Statement

The datasets presented in this study can be found in online repositories. The names of the repository/repositories and accession number(s) can be found in the article/Supplementary Material.

## Author Contributions

D-JG, RS, PS, and Y-RL designed the experiments. D-JG, D-PL, RS, and PS accomplished the experiments. AS, KV, YQ, QK, ZL, X-PS, and MM analyzed the data. D-JG, RS, and PS drafted the manuscript. Y-XX and Y-RL critically revised and finalized the article. All authors reviewed the article and approved it for publication.

## Conflict of Interest

The authors declare that the research was conducted in the absence of any commercial or financial relationships that could be construed as a potential conflict of interest.

## Publisher’s Note

All claims expressed in this article are solely those of the authors and do not necessarily represent those of their affiliated organizations, or those of the publisher, the editors and the reviewers. Any product that may be evaluated in this article, or claim that may be made by its manufacturer, is not guaranteed or endorsed by the publisher.

## Footnotes

1
www.i-sanger.com

2
http://proteomecentral.proteomexchange.org
