# Supplementary material for: Differential Protein Expression Analysis of Two Sugarcane Varieties in Response to Diazotrophic Plant Growth-Promoting Endophyte Enterobacter roggenkampii ED5
Source: Front Plant Sci. 2021 Nov 23;12:727741. doi: 10.3389/fpls.2021.727741 (PMC8649694; doi:10.3389/fpls.2021.727741)
Supplement: Supplementary file 1 [file Table_1.DOCX]

Supplementary Material





**FIGURE S1** Statistical distribution of proteins and peptides identification information. (A) The distribution of peptide of matching error, (B) peptide number distribution, (C) peptide length distribution, (D) protein molecular weight distribution

**
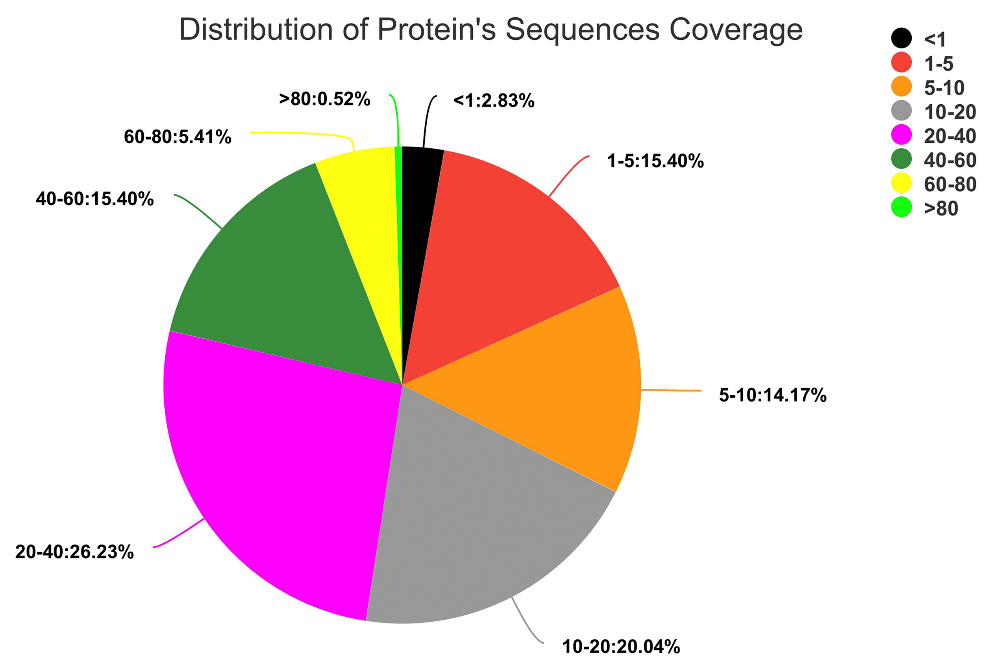
**

**FIGURE S2** Distribution of proteins sequences coverage


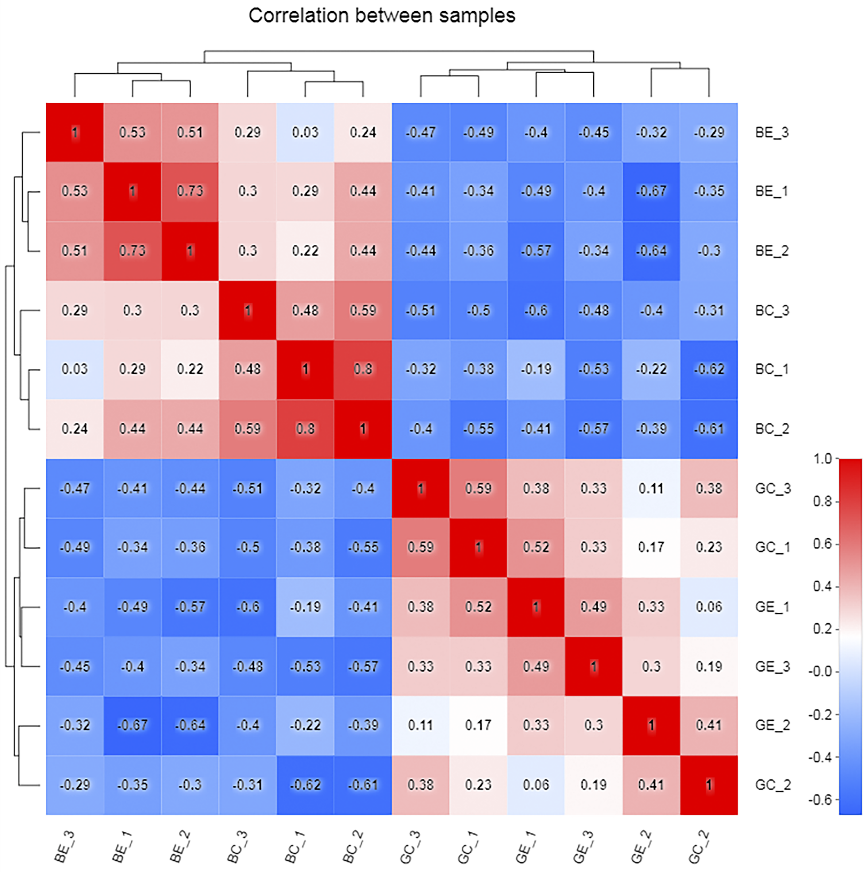


**FIGURE S3** The heatmap of correlation between all samples


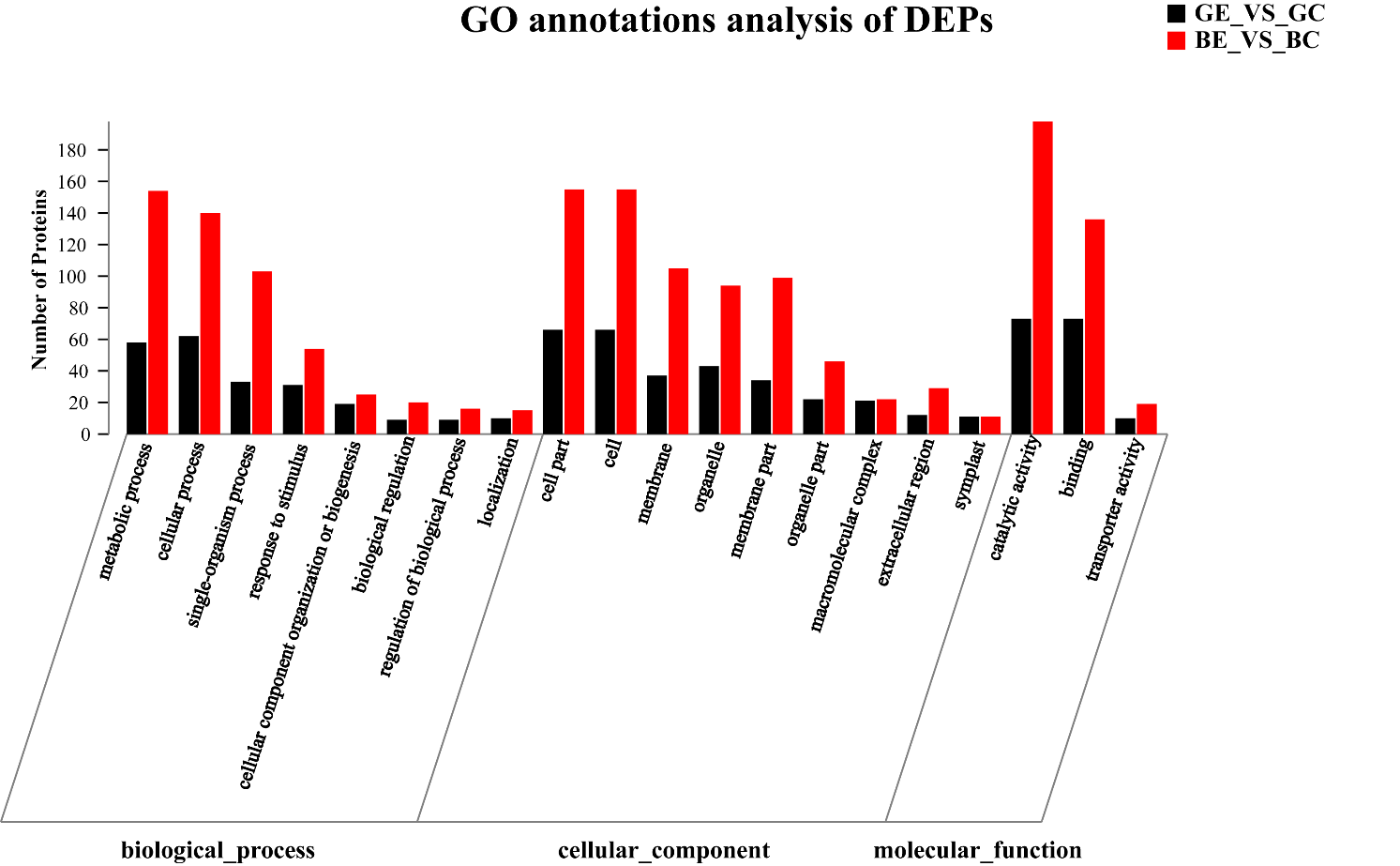
**FIGURE S4** The GO annotations of DEPs in GT11 and B8 sugarcane varieties after inoculation of *E. roggenkampii* ED5

**
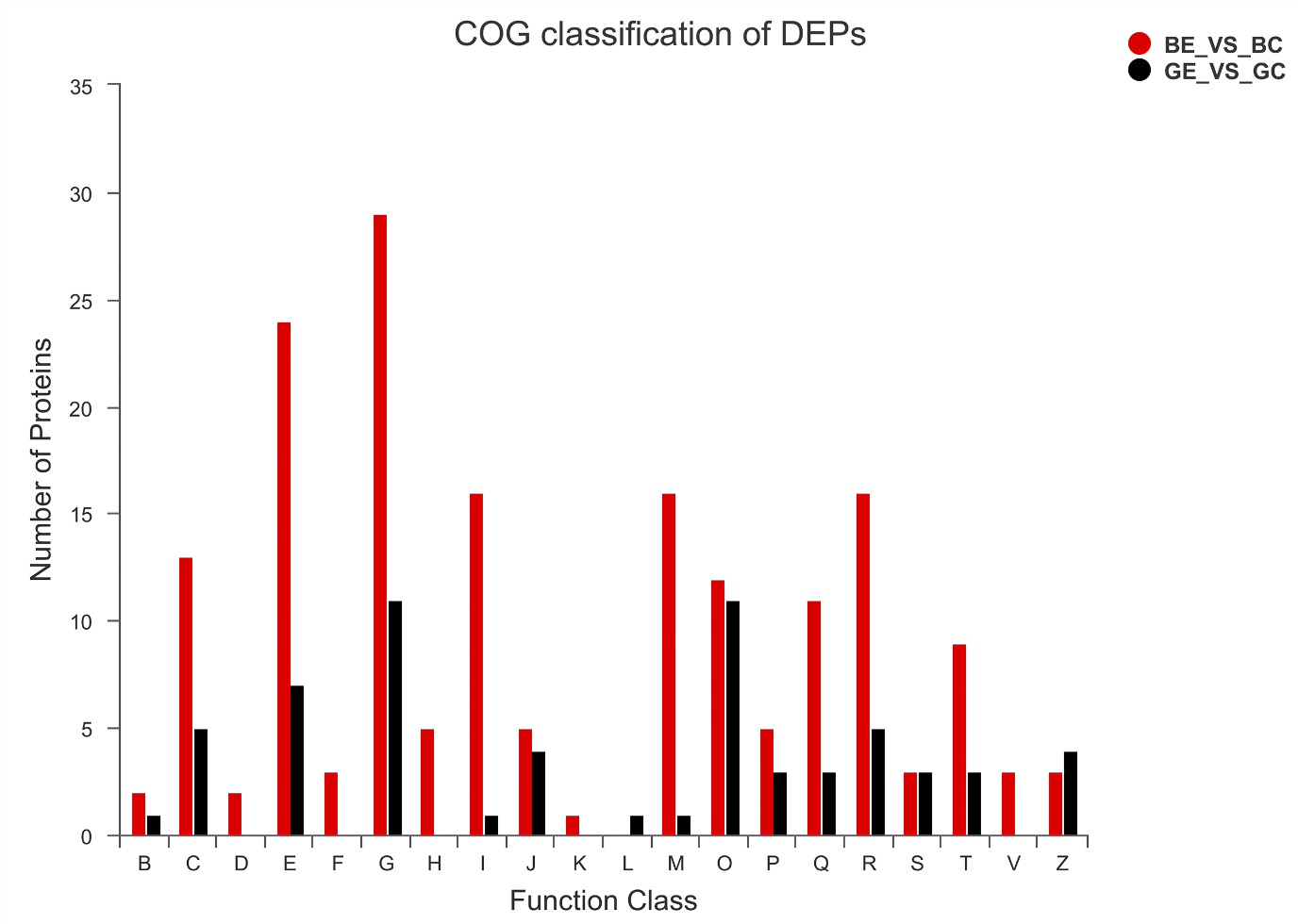
FIGURE S5** The COG annotations of DEPs in GT11 and B8 sugarcane varieties after inoculation of *E. roggenkampii* ED5


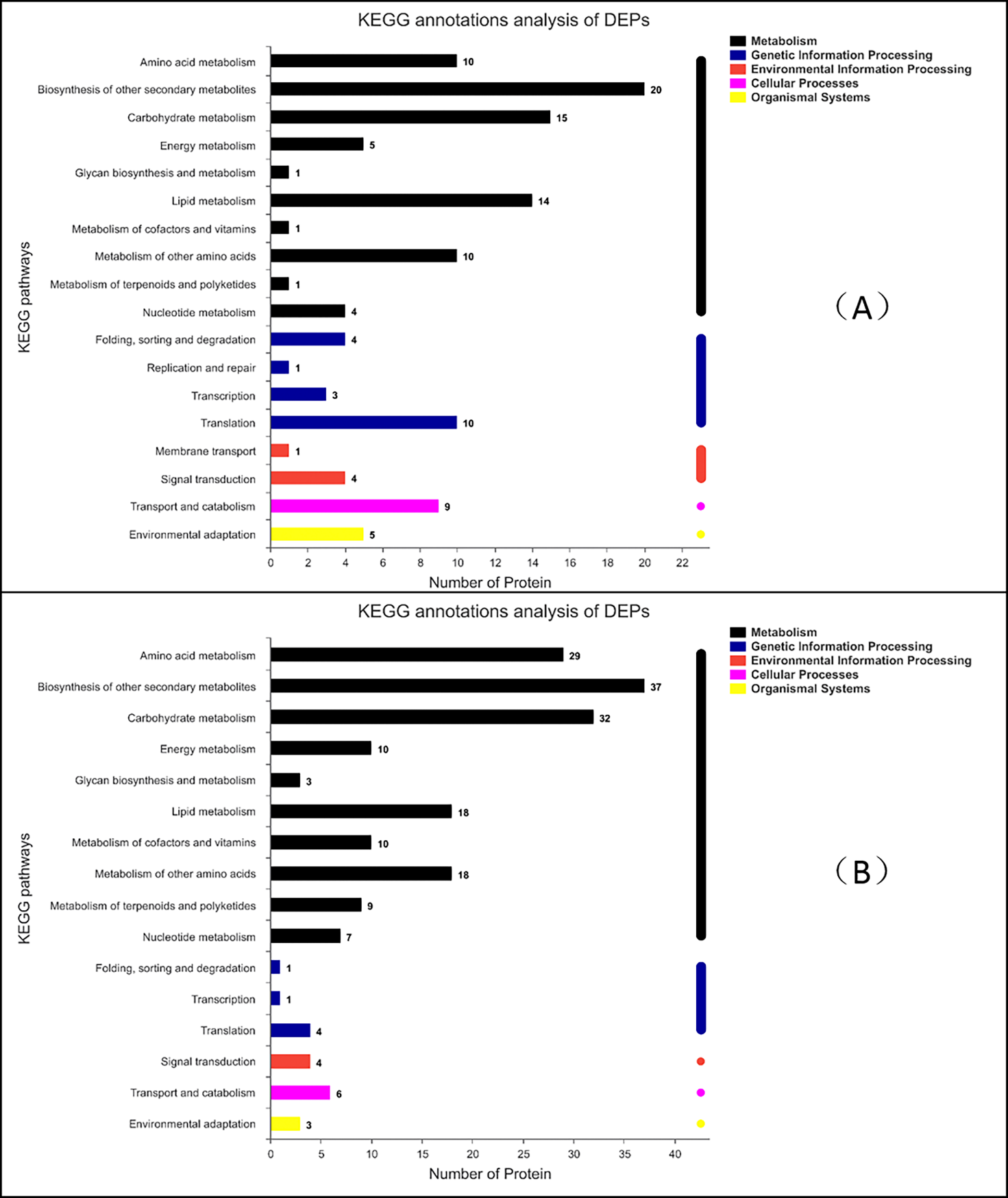
**FIGURE S6** The KEGG annotations of DEPs in (A) GT11 and (B) B8 sugarcane varieties after inoculation of *E. roggenkampii* ED5

**TABLE S1** The results of protein and peptide identification information statistics

| Total Spectrum | Identified Spectrum | Peptide number | Protein number | Protein group number |
| --- | --- | --- | --- | --- |
| 1368062 | 301280 | 73823 | 27508 | 10553 |

**
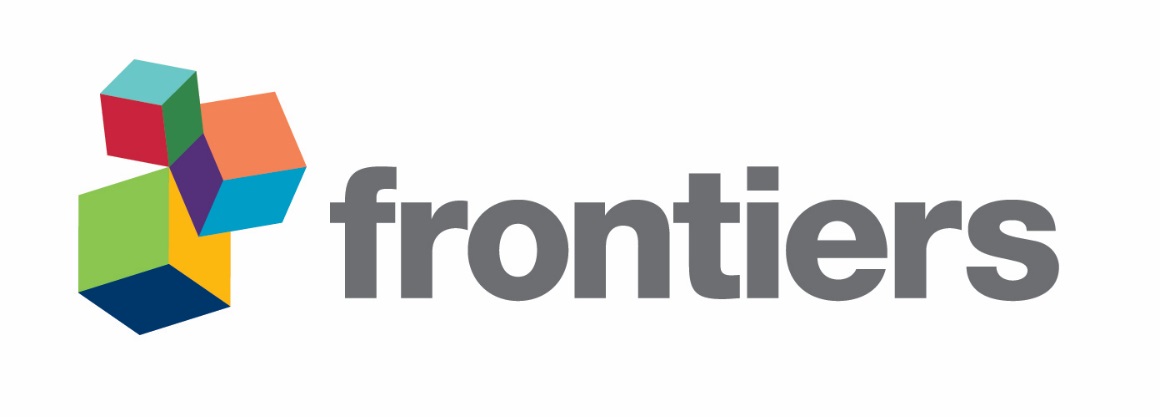
**
